# Multidetector Computed Tomography Angiography for Preoperative Anatomical and Functional Assessment of Living Renal Donors: Correlation With Surgical Findings and Renal Scintigraphy

**DOI:** 10.7759/cureus.106703

**Published:** 2026-04-09

**Authors:** Surya Kant, Anurag Singh, Poonam Verma, Hira Lal, Sunil Kumar, Narayan Prasad, Prasanta K Pradhan

**Affiliations:** 1 Radiodiagnosis, Sanjay Gandhi Post Graduate Institute of Medical Sciences, Lucknow, IND; 2 Pathology, Sanjay Gandhi Postgraduate Institute of Medical Sciences, Lucknow, IND; 3 Opthalmology, Autonomous State Medical College, Lakhimpur Kheri, Lakimpur, IND; 4 Radiodiagnosis, Sanjay Gandhi Postgraduate Institute of Medical Sciences, Lucknow, IND; 5 Nephrology, Sanjay Gandhi Postgraduate Institute of Medical Sciences, Lucknow, IND; 6 Nuclear Medicine, Sanjay Gandhi Postgraduate Institute of Medical Sciences, Lucknow, IND

**Keywords:** angiography, living renal donor, multidetector computed tomography, renal transplant, scintigraphy

## Abstract

Background

Living donor renal transplantation is the most common treatment for end-stage renal disease, and correct donor evaluation is mandatory to ensure safety and improve outcomes. Multidetector computed tomography angiography offers high-resolution anatomical assessment and potential functional evaluation within a single imaging session.

Aims and objectives

The aim and objectives of the study were to analyse multidetector computed tomography accuracy in delineating renal vascular anatomy against surgical findings and compare multidetector computed tomography-derived differential renal function with technetium-99m DTPA (Tc-99m DTPA) scintigraphy.

Material and methods

Sixty-four living renal donors underwent multidetector computed tomography angiography using a triple-bolus contrast protocol, followed by Tc-99m DTPA renal scintigraphy. Renal arterial and venous anatomy, vascular variants, and collecting system abnormalities were evaluated on multidetector computed tomography. Surgical findings were used as the reference standard for vascular anatomy. Differential renal function calculated from computed tomography was compared with scintigraphy using Pearson correlation and Bland-Altman analysis.

Results

Multidetector computed tomography angiography correctly identified renal arteries in 98.44% (63/64) of donors and renal veins in 100% (64) of donors. Sensitivity and specificity for arterial variants were 98% and 100%, respectively, and 100% for venous variants. Multidetector computed tomography-derived differential renal function correlated significantly with scintigraphy-derived differential renal function (right kidney: r=0.59, p<0.01; left kidney: r=0.55, p<0.01) with a mean difference of 0.10% and 95% limits of agreement within ±6% for both kidneys.

Conclusion

Multidetector computed tomography angiography provides the best anatomical detail and shows a moderate agreement with scintigraphy for differential renal function. It is a reliable, comprehensive modality for living donor evaluation in appropriately selected candidates.

## Introduction

Renal transplantation is the choice of treatment for patients with end-stage renal disease, offering better survival and quality of life compared with long-term dialysis [1]. Living donor renal transplantation provides additional advantages, including superior graft survival and reduced waiting times. However, donor safety remains paramount, necessitating fine preoperative evaluation to minimize surgical risk and preserve long-term renal function [2,3].

With the widespread adoption of laparoscopic donor nephrectomy, accurate preoperative delineation of renal vascular anatomy has become increasingly important. The limited operative field and technical complexity of minimally invasive surgery require meticulous knowledge of the number, course, and variants of renal arteries and veins [4]. Failure to identify vascular abnormalities preoperatively can result in intraoperative complications, increased operative time, conversion to an open surgical procedure, or graft injury [4,5].

Traditionally, donor evaluation is based on a combination of excretory urography, digital subtraction angiography, and radionuclide scintigraphy. These investigations are invasive, consume time, and are limited in their ability to examine renal parenchyma and extrarenal abnormalities [6]. The introduction of multidetector computed tomography (MDCT) angiography has revolutionised donor evaluation by using high-resolution isotropic imaging, rapid acquisition, and advanced three-dimensional post-processing [7].

In addition to anatomical assessment, functional evaluation, especially differential renal function (DRF), is a key determinant in screening the kidney for donation. Technetium-99m DTPA (Tc-99m DTPA) renal scintigraphy remains the reference standard for DRF assessment. However, recent literature suggests that MDCT-derived parameters, such as renal volume and contrast enhancement, may give reliable estimates of split renal function, significantly reducing the need for additional nuclear medicine studies [8,9].

The present study was undertaken to evaluate the accuracy of MDCT angiography in delineating renal vascular anatomy by correlating imaging findings with surgical observations and to assess the agreement between MDCT-derived DRF and Tc-99m DTPA renal scintigraphy in living renal donors.

## Materials and methods

Study design and population

This prospective observational study was conducted in the Department of Radiodiagnosis at a tertiary care center after approval from the institutional ethics committee. Voluntary living renal donors were recruited from the renal transplant programme between November 2011 and June 2013. Written informed consent was obtained from all participants. We had included voluntary living renal donors aged 18-years or older. The donors who had refused consent, were pregnant or lactating, had serum creatinine greater than 1.6 mg/dL, and had known contraindications to iodinated contrast media were excluded from the study. A total of 64 donors met the inclusion criteria and were enrolled in the present study.

Multidetector computed tomography angiography protocol

MDCT angiography was performed using a 64-slice multidetector CT scanner. The unenhanced CT acquisition was extended from above the kidney to the pubic symphysis with breath hold in inspiration. A triple-bolus contrast administration protocol was used to obtain combined arterial, venous, and nephrographic phase images in a single acquisition, followed by a delayed excretory phase. This protocol was designed to optimise vascular visualisation while limiting radiation exposure.

**Figure 1:**
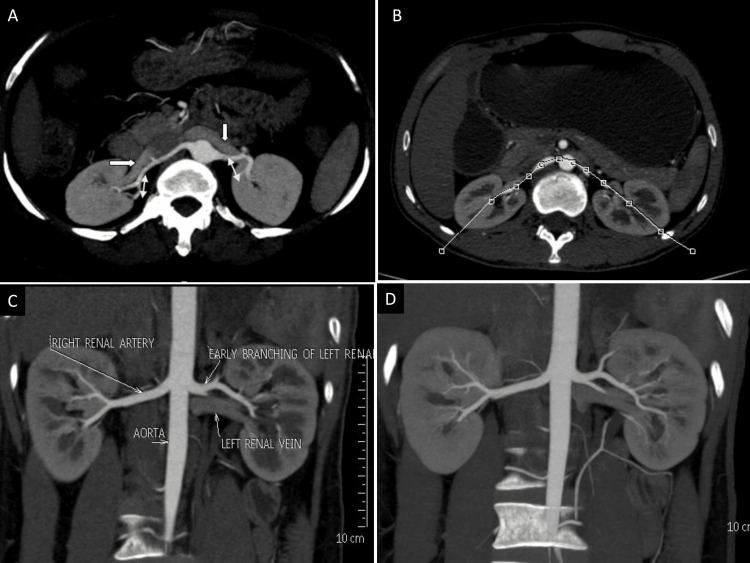
Computed tomography angiographic evaluation of renal arterial anatomy (A) Post-processing axial maximum intensity projection image showing bilateral renal veins (→) lying anterior to renal arteries (⇔)(B) Axial computed tomography image displaying curved lined drawn at the level of renal artery to create a curved plane (c) Curved coronal reformatted image showing bilateral single renal artery with early branching of left renal artery (D) Curved maximum intensity projection image displaying renal arteries branches better.

Post-processing was performed using axial images, multiplanar reformations, maximum intensity projections, and volume-rendered images. The renal arteries were evaluated for their number, origin, and early division, which is defined as branching occurring within 1.5 cm of the ostium (Figure 1).

**Figure 2:**
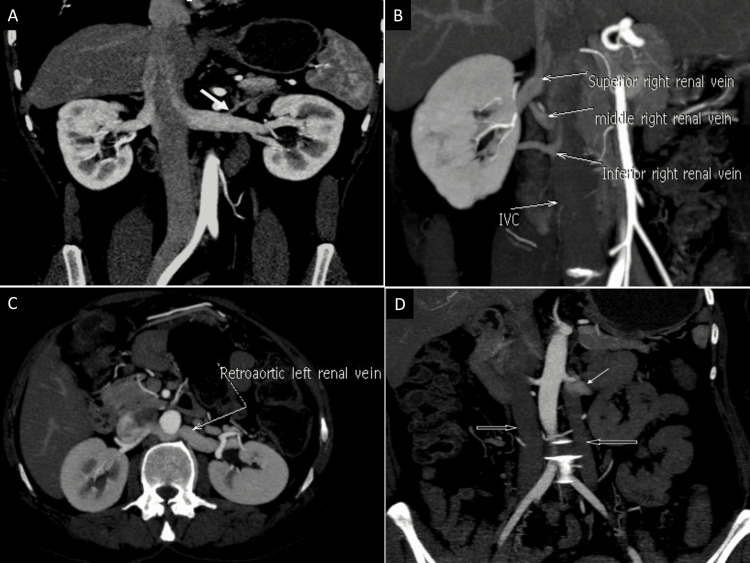
Computed tomography angiographic depiction of renal venous anatomy (A) Post-processing curved coronal reformatted image showing bilateral single renal vein with left adrenal vein draining in to left renal vein (→)  (B) Coronal oblique maximum intensity projection image displaying multiple right renal veins (C) Coronal oblique maximum intensity projection image showing retro aortic left renal vein (D) Coronal maximum intensity projection image displaying double inferior vena cava (⇒) with left inferior vena cava joining left renal vein (→).

The renal veins were examined for number, confluence configuration, and significant variations, including circum-aortic or retro-aortic left renal veins and duplication of the inferior vena cava (Figure 2).

Collecting system abnormalities and incidental renal or extrarenal findings were also recorded.

Differential renal function assessment

Renal volume of both kidneys was calculated by software semiautomatically with manual correction, and the renal pelvis and fat were excluded. The remaining structure was selected to measure the volume (V). Mean attenuation of the kidney was taken on the unenhanced images and subtracted from the mean attenuation taken at the same level in contrast-enhanced images to give the enhancing attenuation of the kidney (A). Volume and enhancing attenuation were computed for each kidney in all patients. Right DRF (rDRF) was then calculated as the product of volume and attenuation for the right kidney divided by the sum of the product of volume and attenuation for both kidneys.

 rDRF = [rV x rA]/ [rV x rA] + [lV x lA]

 r= right, l= left, V= Volume, A= Attenuation, DRF= Differential Renal Function

Likewise, left DRF (lDRF) was also calculated. All donors underwent Tc-99m DTPA renal scintigraphy using standard protocols. 

Statistical analysis

Surgical findings during donor nephrectomy served as the reference standard for anatomical assessment. Sensitivity, specificity, positive predictive value, and overall accuracy of MDCT angiography were calculated. The Pearson correlation coefficient was used to assess the relationship between MDCT-derived and scintigraphy-derived DRF. Agreement between the two methods was evaluated using Bland-Altman analysis. Statistical analysis was performed using SPSS version 26.0 (IBM Corp, Armonk, USA).

## Results

All 64 potential renal donors underwent CT angiography; this imaging was performed only after completion of the standard pre-donation assessment protocol. This included essential clinical, biochemical, and preliminary radiological assessments to confirm donor suitability before selection for advanced imaging. After undergoing CT angiography, renal graft harvesting was performed in all donors. The mean age was 44.5 years (range: 22-66 years). Left donor nephrectomy was performed in 50 (78.12%) donors, while right nephrectomy was performed in 14 (21.88%) donors. The right kidney was selected primarily due to unfavourable vascular anatomy on the left side (Table 1).

**Table 1:** Demographic profile and side of donor nephrectomy (n=64)

Variable	Value
Mean age (years)	44.5
Left nephrectomy	50 (78.12%)
Right nephrectomy	14 (21.88%)
Laparoscopic nephrectomy	38 (59.37%)
Open nephrectomy	26 (40.63%)

Left nephrectomy predominated due to the longer renal vein and more favourable venous anatomy. Right nephrectomy was reserved for cases with left-sided vascular variants, highlighting the importance of correct preoperative imaging.

Renal arterial and venous anatomy

MDCT angiography successfully identified renal arterial and venous anatomy in most of the donors. Multiple renal arteries were present in 15 (23.44%) of right kidneys and 17 (26.56%) of left kidneys. Venous multiplicity was more common on the right side, while complex venous variants were confined to the left side (Table 2).

**Table 2:** Renal arterial and venous anatomy on multidetector computed tomography angiography

Anatomy	Left kidney	Right kidney
Renal arteries		
Single artery	49 (77%)	47 (74%)
Two arteries	14 (22%)	15 (23%)
Three arteries	1 (1%)	2 (3%)
Renal veins		
Single vein	58 (91%)	48 (75%)
Two veins	6 (9%)	14 (22%)
Three veins	0	2 (3%)

Right-sided venous multiplicity was common and is clinically relevant due to increased technical difficulty during donor nephrectomy.

Correlation with surgical findings

MDCT angiography findings were compared with intraoperative observations with a confidence interval of 95%. A small-calibre auxiliary renal artery (<3 mm) was overlooked during the initial MDCT interpretation but was subsequently detected in a retrospective analysis. All renal veins and their venous variations were precisely identified before surgery (Table 3).

**Table 3:** Diagnostic performance of multidetector computed tomography angiography compared with surgical findings

Parameter	Renal arteries	Renal veins
Sensitivity (confidence interval-95%)	98%	100%
Specificity (confidence interval-95%)	100%	100%
Positive predictive value	100%	100%
Overall accuracy	98%	100%

MDCT angiography showed superior diagnostic efficacy, especially regarding venous structure. The missed accessory artery did not alter surgical management, highlighting the clinical adequacy of MDCT angiography.

Differential renal function

Mean scintigraphy-derived split renal function was nearly symmetrical between both kidneys. MDCT-derived DRF showed moderate correlation with scintigraphy results (Table 4).

**Table 4:** Comparison of multidetector computed tomography-derived and Tc-99m DTPA-derived differential renal function

Parameter	Left kidney	Right kidney
Mean DRF by scintigraphy (%)	50.0±3.4	49.9±3.4
Mean DRF by MDCT (%)	49.9±3.4	50.0±3.4
Pearson correlation (r)	0.55	0.59
Mean difference (%)	0.1	0.1
95% limits of agreement (%)	−5.8 to 5.6	−5.5 to 5.8

**Figure 3:**
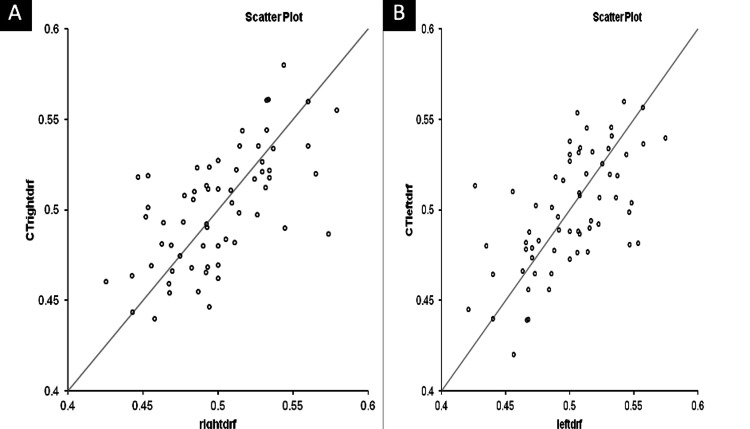
Scatter Plot displaying correlation between MDCT-derived and scintigraphy-derived differential renal function (A&B) Pearson correlation demonstrated a statistically significant positive correlation for both kidneys.

Scatter plots showing positive correlation between computed tomography-derived DRF and renal scintigraphy-derived DRF of the right kidney (Figure 3A) and left kidney (Figure 3B) with a line of equality (p<0.001).

**Figure 4:**
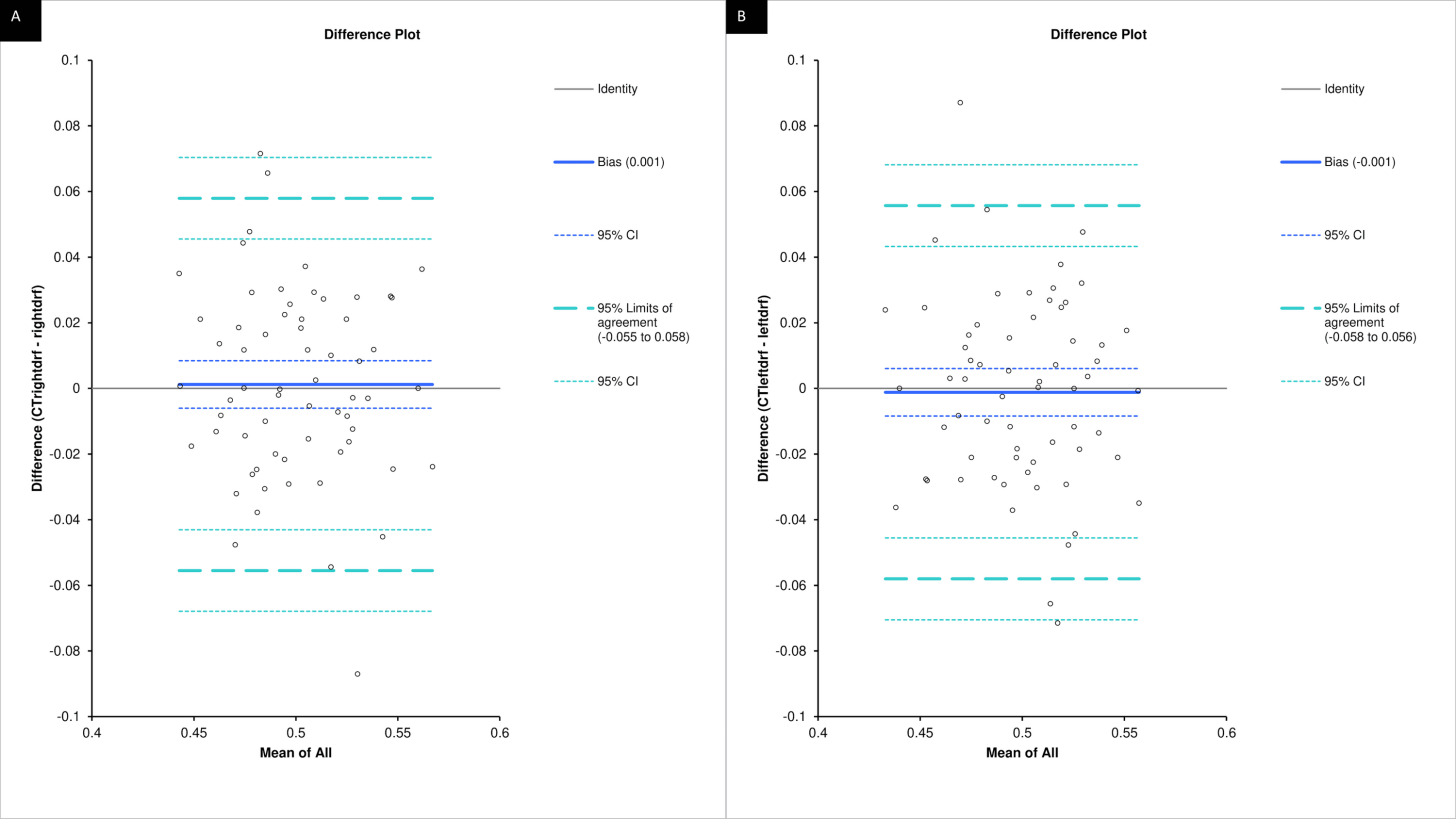
Bland–Altman analysis showing agreement between MDCT and scintigraphy for renal function) (A&B) The Bland–Altman analysis demonstrated negligible bias and acceptable limits of agreement for both kidneys.

Bland-Altman analysis showed the difference between computed tomography and radionuclide renography-based determinations of split function in relation to the mean difference of the right kidney and the 95% confidence interval (CI) for the difference score data set (dashed lines) (y=±1.96 SD). Right MDCT-derived DRF and Tc-99 m DTPA scintigraphy shows good agreement, as 95% limits of agreement are small (−5.5% to 5.8%) (Figure 4A). Bland-Altman analysis showed the difference between computed tomography and radionuclide renography-based determinations of split function in relation to the mean difference of the left kidney and the 95% CI for the difference score data set (dashed lines) (y=±1.96 SD). Left MDCT-derived DRF and Tc-99 m DTPA scintigraphy shows good agreement, as 95% of the limits of agreement are small (−5.8% to 5.6%) (Figure 4B).

These findings indicate a moderate correlation between MDCT-derived and scintigraphy-derived DRF, with differences unlikely to affect clinical decision-making among donors exhibiting nearly symmetrical renal function.

## Discussion

Living donor renal transplantation remains the best treatment modality for end-stage renal disease, offering superior patient survival, graft longevity, and quality of life compared with long-term dialysis. As the donor is healthy, the evaluation process first prioritises donor safety while ensuring the best graft selection [10]. Radiological examination plays a central role in this evaluation process by providing critical structural and functional information mandatory for surgical planning. This research underscores the significance of MDCT angiography as a comprehensive and effective investigation method for the preoperative assessment of living renal donors [11,12].

Importance of accurate vascular mapping

Donor nephrectomy progressively uses laparoscopic and robotic-assisted methods, making renal vascular structural anatomy identification crucial. Insufficient tactile feedback and limited operational fields make vascular alterations a key intraoperative issue. This research found that MDCT angiography accurately delineated renal artery and venous architectural difficulties, matching near-surgical findings [13].

Anatomical studies predict that 25%-35% of patients have auxiliary renal arteries, which is in concordance with our study. The damage or closure of these terminal arteries, which specifically serve renal segments, may cause segmental infarction or postoperative graft failure [14]. MDCT angiography capability to preoperatively identify these arteries enables urosurgeons to adjust their surgical strategy, choose the suitable kidney for donation, or devise vascular repair when required [14,15].

Although a small accessory artery was missed in our study, it was of minimal calibre and did not influence the surgical outcome. This study highlights a significant clinical consideration: although the absolute detection of every minute vessel may not always be attainable, MDCT consistently detects anatomies pertinent to surgery. Similar findings have been reported in the literature, where missed vessels are typically small (<2-3 mm) and rarely affect graft viability or donor safety [16,17].

Venous anatomy and surgical relevance

Renal venous anatomy is often more variable and complex than arterial anatomy, particularly on the right side. Multiple renal veins, reduced length, and anomalous drainage patterns increase technical difficulty during donor nephrectomy. In the present study, MDCT angiography accurately identified all venous variants, including circumaortic and retroaortic left renal veins and duplicated inferior vena cava [13,18].

This perfect concordance with surgical findings highlights one of the best advantages of MDCT angiography. Preoperative recognition of venous anomalies allows urosurgeons to anticipate challenges, select optimal dissection planes, and reduce the risk of intraoperative haemorrhage [19]. Several studies have demonstrated that unrecognised venous variants are a leading cause of conversion from laparoscopic to open nephrectomy procedures. Findings of the present study support the routine use of MDCT angiography as the primary tool for venous mapping in donor evaluation [19,20].

Kidney selection and laterality

In most transplants, the left kidney is preferred for donation due to its longer renal vein and more favourable venous anatomy. This selection preference was reflected in our cohort, where 50 (78.12%) of donors underwent left nephrectomy. Right nephrectomy was reserved for cases with unfavourable left-sided anatomy, such as multiple arteries or complex venous anatomy [21].

MDCT angiography played a crucial role in guiding these decision-making paradigms. By providing a detailed comparison of bilateral anatomy, it enabled objective selection of the kidney that would maximise recipient benefit while preserving donor safety. This individual-tailored approach is most important in donors with asymmetrical anatomy or borderline functional differences [21,22].

Functional assessment: expanding the role of MDCT

Assessment of DRF is a cornerstone of donor evaluation. The guiding principle is to leave the donor with the kidney that has better function, thereby minimising the risk of future renal insufficiency. Traditionally, radionuclide scintigraphy using Tc-99m DTPA or MAG3 has been considered indispensable for this purpose [23,24].

In recent years, there has been growing interest in deriving functional information from MDCT datasets. Renal volume is a surrogate marker of nephron mass, while contrast enhancement reflects renal perfusion and parenchymal function. Several investigators have demonstrated moderate to strong correlations between renal volume and split renal function measured by scintigraphy [25,26].

In the present study, MDCT-derived DRF showed a moderate positive correlation with scintigraphy-derived DRF for both kidneys. Bland-Altman analysis demonstrated minimal bias and narrow limits of agreement, suggesting that the two methods are showing positive correlation. The observed differences were less likely to influence kidney selection in donors with nearly symmetrical function, which represents the majority of healthy donors [26,27]. These findings corroborate the expanding evidence that MDCT can provide dependable functional assessments and can be used in addition to nuclear medicine investigations [26-28].

When scintigraphy retains significance

Notwithstanding these encouraging findings, MDCT-derived functional evaluation should not be regarded as a comprehensive substitute for radionuclide scintigraphy in each case. Donors exhibiting considerable functional asymmetry, limited renal function, previous renal disease, or congenital defects may necessitate scintigraphic validation. Additionally, scintigraphy provides direct physiological measurement of renal filtration, which is not completely captured by CT-based parameters [28,29].

Radiation dose considerations

Radiation exposure is a critical concern in healthy donors. The triple-bolus protocol used in this study was designed to optimise vascular visualisation while minimising radiation dose by combining arterial and venous information into a single acquisition. With modern scanners, dose-reduction techniques such as automated tube current modulation, low-kilovoltage imaging, and iterative reconstruction have further reduced radiation exposure [29,30].

When compared with the cumulative radiation dose from multiple investigations, including conventional angiography, excretory urography, and scintigraphy-MDCT angiography, it represents a favourable balance between diagnostic yield and radiation risk. Continuous protocol optimisation remains essential to uphold the principle of “as low as reasonably achievable” (ALARA) [30,31].

Incidental findings and added value of MDCT

An additional advantage of MDCT angiography is its ability to detect incidental renal and extrarenal findings. Small renal cysts, calculi, parenchymal scars, and unsuspected extrarenal abnormalities may influence donor eligibility or surgical planning. Early detection of such findings contributes to donor safety and may prevent postoperative complications [32].

Limitations of the study

The present study has certain limitations, as it was conducted at a single tertiary care center with a relatively modest sample size. Functional assessment by MDCT is indirect and relies on surrogate markers rather than direct physiological measurement. Long-term donor outcomes were not assessed, which would further validate the clinical impact of MDCT-based evaluation.

## Conclusions

MDCT angiography is accurate, non-invasive, and comprehensive for preoperative evaluation of living renal donors. It matched surgical vascular findings and showed moderate agreement with Tc-99m DTP. MDCT can be the most common imaging modality for evaluating living kidney donors, although it is integrated with radioactive studies. Future MDCT functional evaluation studies, by including bigger multicentric cohorts and longer follow-up, may further validate the results. Dual-energy CT, perfusion imaging, and artificial intelligence-driven volumetric analysis could improve functional evaluation and minimise the need for additional testing.
